# Infants with Congenital Disorders Identified Through Newborn Screening — United States, 2015–2017

**DOI:** 10.15585/mmwr.mm6936a6

**Published:** 2020-09-11

**Authors:** Marci K. Sontag, Careema Yusuf, Scott D. Grosse, Sari Edelman, Joshua I. Miller, Sarah McKasson, Yvonne Kellar-Guenther, Marcus Gaffney, Cynthia F. Hinton, Carla Cuthbert, Sikha Singh, Jelili Ojodu, Stuart K. Shapira

**Affiliations:** ^1^Center for Public Health Innovation, CI International, Littleton, Colorado; ^2^Department of Epidemiology, Colorado School of Public Health, University of Colorado Denver, Anschutz Medical Campus, Aurora, Colorado; ^3^Association of Public Health Laboratories, Silver Spring, Maryland; ^4^National Center on Birth Defects and Developmental Disabilities, CDC; ^5^Department of Community and Behavioral Health, Colorado School of Public Health, University of Colorado Denver, Anschutz Medical Campus, Aurora, Colorado; ^6^Newborn Screening and Molecular Biology Branch, Division of Laboratory Sciences, CDC.

Newborn screening (NBS) identifies infants at risk for congenital disorders for which early intervention has been shown to improve outcomes (*1*). State public health programs are encouraged to screen for disorders on the national Recommended Uniform Screening Panel (RUSP), which increased from 29 disorders in 2005 to 35 in 2018.* The RUSP includes hearing loss (HL) and critical congenital heart defects, which can be detected through point-of-care screening, and 33 disorders detected through laboratory screening of dried blood spot (DBS) specimens. Numbers of cases for 33 disorders on the RUSP (32 DBS disorders and HL) reported by 50 U.S. state programs were tabulated. The three subtypes of sickle cell disease (SCD) listed as separate disorders on the RUSP (S,S disease; S,beta-thalassemia; and S,C disease) were combined for the current analysis, and the frequencies of the resulting disorders were calculated relative to annual births. During 2015–2017, the overall prevalence was 34.0 per 10,000 live births. Applying that frequency to 3,791,712 live births in 2018,^†^ approximately 12,900 infants are expected to be identified each year with one of the disorders included in the study. The most prevalent disorder is HL (16.5 per 10,000), and the most prevalent DBS disorders are primary congenital hypothyroidism (CH) (6.0 per 10,000), SCD (4.9 per 10,000), and cystic fibrosis (CF) (1.8 per 10,000). Notable changes in prevalence for each of these disorders have occurred since the previous estimates based on 2006 births (*2*). The number of infants identified at a national level highlights the effect that NBS programs are having on infant health through early detection, intervention, and potential improved health, regardless of geographic, racial/ethnic, or socioeconomic differences.

A 2008 report estimated that in 2006, 6,439 U.S. infants were identified with any of 27 DBS disorders included on the RUSP (*2*). Because complete data were available from only four states, that estimate was derived from nonlinear modeling techniques applied to 2001–2006 NBS data reported by those states, extrapolated to 2006 U.S. births adjusted for the race/ethnicity distributions (*2*). The objectives of the current study were to update national estimates of infants with NBS disorders included on the RUSP and to compare these updated prevalence estimates with those previously reported.

The current study is based on data reported for 33 of the 35 disorders included on the RUSP among infants born during 2015–2017, the most recent years of available national data. States reported aggregate numbers of confirmed cases of 32 RUSP DBS disorders to the Association of Public Health Laboratories’ Newborn Screening Technical assistance and Evaluation Program (NewSTEPs) (*3*), a Health Resources and Services Administration-funded data repository. States were requested to apply uniform case definitions established by clinical and public health experts and adopted by NewSTEPs (*4*). All 50 state programs reported data to NewSTEPs; however, several states were unable to report data for some of the DBS disorders included in this study. In addition, four disorders (severe combined immunodeficiency, glycogen storage disease type II [Pompe disease], mucopolysaccharidosis type 1, and X-linked adrenoleukodystrophy) were added to the RUSP since 2006, for which screening was implemented in some states during the 3-year data collection time frame. Aggregate numbers of confirmed cases of HL among 2015–2017 births were reported from 48 states to CDC’s Early Hearing Detection and Intervention (EHDI) Hearing Screening and Follow-up Survey (HSFS) (*5*).^§^ Colorado did not report HL data for the 2015–2017 period, and Minnesota reported data for 2017 only. The District of Columbia did not report data for any of the DBS NBS disorders, so it was excluded from the analysis.

Because SCD is generally considered a condition comprising multiple subtypes,^¶^ the three SCD subtypes on the RUSP were combined into a single disorder for the current assessment. Two RUSP disorders were not included in this assessment: critical congenital heart defects, because few states require reports (*6*), and spinal muscular atrophy, because it was not added to the RUSP until after the 2015–2017 period covered in this study.**

Annual births for each state during 2015–2017 and nationally in 2018 (the most recent year with data) were ascertained from CDC WONDER.^††^ For each of the disorders, prevalence estimates for 2015–2017 were calculated. For each disorder, denominator data included only births during months for which universal screening was available in the state and the state reported data for the disorder to NewSTEPs or HSFS. To estimate annual case counts, the 2015–2017 prevalence rates calculated empirically in this assessment were applied to the total U.S. birth cohort for 2018. Prevalence estimates for NBS disorders among infants born during 2015–2017 were compared with estimates among infants born in 2006. For 27 DBS disorders on the RUSP, 2006 estimates were ascertained from the NBS modeling study report (*2*). These data were supplemented with HL data reported to HSFS for 2006.

Prevalence estimates for each of the disorders and all disorders combined are presented for all 50 states (Table), and the prevalence of each disorder is presented by state in a heat map (Supplementary Table, https://stacks.cdc.gov/view/cdc/93107). During 2015–2017, the birth prevalences for any of the disorders, any of the DBS disorders, and HL were estimated at 34.0, 17.5, and 16.5 per 10,000, respectively. Prevalences of individual DBS disorders varied from 0.01 to 6.0 per 10,000. The most prevalent DBS disorders were CH (6.0 per 10,000), SCD (4.9 per 10,000), and CF (1.8 per 10,000); together, these accounted for 73% of all cases of DBS disorders.

**TABLE:** Aggregate newborn screening disorder frequency, prevalence, and expected cases compared with modeled 2006 data for selected disorders, based on frequencies reported in four states, 2001–2006* — 50 state NBS programs, United States, 2015–2017

Disorder	No. of cases reported 2015–2017^†^	No. of births^§^	Rate (cases per 10,000 births)	2006 modeled rate*	Rate difference	Expected no. of cases per year^¶^
**Amino acid disorders**						
Classical phenylketonuria and hyperphenylalaninemia	691	11,750,876	0.59	0.52	0.07	223
Maple syrup urine disease	64	11,750,876	0.05	0.06	−0.01	21
Homocystinuria	18	11,750,876	0.02	0.03	−0.01	6
Citrullinemia, type I	75	11,750,876	0.06	0.06	0.01	24
Argininosuccinic aciduria	59	11,750,876	0.05	0.02	0.03	19
Tyrosinemia, type I	22	11,750,876	0.02	NR*	—*	7
**Organic acid disorders**						
Isovaleric acidemia	84	11,750,876	0.07	0.08	−0.01	27
Glutaric acidemia, type I	104	11,750,876	0.09	0.09	−0.00	34
3-Hydroxy-3-methylglutaric aciduria	6	11,750,876	0.01	0.01	−0.00	2
3-Methylcrotonyl-CoA carboxylase deficiency	293	11,750,876	0.25	0.24	0.01	95
Methylmalonic acidemia (methylmalonyl-CoA mutase)	22	11,750,876	0.02	0.12	−0.10	7
Propionic acidemia	63	11,750,876	0.05	0.04	0.02	20
Methylmalonic acidemia (cobalamin disorders)	43	11,750,876	0.04	0.03	0.01	14
Holocarboxylase synthase deficiency	6	11,750,876	0.01	0.01	−0.00	2
β-Ketothiolase deficiency	8	11,750,876	0.01	0.02	−0.01	3
**Fatty acid oxidation disorders**						
Medium-chain acyl-CoA dehydrogenase deficiency	689	11,750,876	0.59	0.58	0.01	222
Very long-chain acyl-CoA dehydrogenase deficiency	206	11,750,876	0.18	0.17	0.01	66
Long-chain L-3 hydroxyacyl-CoA dehydrogenase deficiency	26	11,750,876	0.02	0.03	−0.01	8
Trifunctional protein deficiency	6	11,750,876	0.01	0.00	0.00	2
Carnitine uptake defect/carnitine transport defect	138	11,750,876	0.12	0.21	−0.09	45
**Hemoglobinopathies**						
SCD (includes S,S disease, S,beta-thalassemia, and S,C disease)	5,808	11,750,876	4.94	4.29	0.65	1,874
**Endocrine disorders**						
Primary congenital hypothyroidism	6,629	11,049,582	6.00	5.21	0.79	2,275
Congenital adrenal hyperplasia	819	11,750,876	0.70	0.49	0.21	264
**Lysosomal storage disorders**						
Glycogen storage disease, type II (Pompe)	62	1,828,917	0.34	—**	—**	129
Mucopolysaccharidosis, type 1	11	965,027	0.11	—**	—**	43
**Other DBS screening disorders**						
Biotinidase deficiency	477	11,750,876	0.41	0.15	0.26	154
Cystic fibrosis	2,145	11,750,876	1.83	3.02	−1.19	692
Classical galactosemia	249	11,750,876	0.21	0.54	−0.33	80
Severe combined immunodeficiencies	220	9,763,119	0.23	—**	—**	85
X-linked adrenoleukodystrophy	83	1,561,394	0.53	—**	—**	202
**Point-of-care screening disorders^††^**						
Hearing loss	19,167	11,611,293	16.51	9.90^§§^	6.61	6,259
**Infants expected to be detected with an NBS disorder**						12,905
Prevalence per 10,000 births						34.0
**Infants expected to be detected via DBS screening**						6,646
Prevalence per 10,000 births, DBS only						17.5

**Abbreviations:** DBS = dried blood spot; NBS = newborn screening; NR = not reported; SCD = sickle cell disease.

* https://www.cdc.gov/mmwr/preview/mmwrhtml/mm5737a2.htm. Tyrosinemia, type I was not included because of unreliable data at the time of the report.

^†^ Data were not available for the following disorders and states: primary congenital hypothyroidism from New York (2015**–**2017) and hearing loss from Colorado (2015–2017) and Minnesota (2015, 2016).

^§^ The number of births includes only births that occurred during 2015–2017 that each state conducted screening for the disorder and reported data to the Association of Public Health Laboratories, Newborn Screening Technical assistance and Evaluation Program or CDC’s Hearing Screening and Follow-up Survey.

^¶^ Disorder frequency based on 3,791,712 live births nationally (50 states and the District of Columbia [DC]) in 2018; all case numbers are rounded estimates.

** Not included on the Recommended Uniform Screening Panel in 2006.

**^††^** State level data for critical congenital heart defects, the other point-of-care screen on the Recommended Uniform Screening Panel, are not included in this table as data are not available from most states despite universal screening in the United States for these disorders.

^§§^ Prevalence based on hearing loss cases reported by 45 states and DC in 2006 to CDC’s Hearing Screening and Follow-up Survey.

The estimated 2006 prevalence of any DBS disorder on the RUSP, other than type 1 tyrosinemia, was 15.6 per 10,000 (6,439 per 4,138,349) births (*2*). The estimated number of HL cases in 2006 based on HSFS data was 4,097 (9.9 per 10,000) (Table). Thus, the total 2006 prevalence estimate for any of the assessed disorders on the RUSP was 25.5 per 10,000 infants. The RUSP disorders prevalence estimate of 34.0 per 10,000 reported here for infants born during 2015–2017 is a 33% increase since 2006, with more than three-quarters (78%) of that increase driven by HL.

Notable changes in prevalence between 2006 and 2015–2017 occurred for several disorders. Among the more prevalent DBS disorders, the observed rate during 2015–2017 was lower than the modeled rate in 2006 for CF (−1.19 per 10,000) and higher for SCD (0.65 per 10,000) and CH (0.79 per 10,000). The observed rate for HL during 2015–2017 was substantially higher (16.5 per 10,000) than the rate based on 2006 HSFS data (9.9 per 10,000). Variable prevalences by state were observed, with HL, CH, and SCD being the most prevalent in most states (Supplementary Table, https://stacks.cdc.gov/view/cdc/93107).

Applying the 2015–2017 prevalence estimate of 34.0 per 10,000 live births to the number of U.S. live births in 2018 (3,791,712), approximately 12,900 infants (6,646 with DBS disorders and 6,259 with HL) are expected to be identified annually with one of the included NBS disorders.

## Discussion

This is the first published report of the prevalence of NBS disorders in the United States using cases reported by all 50 states. Based on 2018 live births, approximately 12,900 U.S. infants are predicted to be identified each year through NBS with one of the included RUSP disorders (DBS and HL). This total reflects only a modest increase of 3.2% in the number of infants identified with a DBS disorder between 2006 (6,439 infants) and the expected number in 2018 (6,646 infants), even though four new disorders with an estimated 459 infants identified in 2018 (7.1% increase) were added to the RUSP since 2006. This small increase in the number of reported cases is less than one half of the expected increase from the new disorders if the number of births had remained the same. It is the net result of an increase in the prevalence of identified infants with DBS disorders since 2006 for both existing and new RUSP disorders and a marked reduction in the number of births in the United States after 2006. In contrast, the number of infants identified with HL increased substantially from an estimated 4,097 in 2006 to an expected 6,259 in 2018; this large increase likely reflects improvements in follow-up documentation by EHDI programs (*5*).

Although the overall prevalence of DBS disorders increased from 2006 (15.6 per 10,000) to 2015–2017 (17.5 per 10,000), changes in individual disorder prevalence estimates varied. Random variation and small numbers might have affected the estimates for each period. Notable changes in prevalence for each of the three most prevalent DBS disorders were observed. First, the lower prevalence of CF during 2015–2017 compared with 2006 might reflect a reduction in live births with CF under the influence of widespread neonatal and prenatal screening and reproductive counseling (*7*). Second, the increase in CH prevalence might be a continuation of long-term trends related to a higher proportion of U.S. births to Hispanic parents, among other factors (*8*). Finally, the higher prevalence of SCD might reflect more births to parents originating from countries where SCD is relatively common^§§^ (*9*). Variations in prevalence of individual disorders across states might reflect differences in the geographic distribution of disease-causing genetic variants and differences in screening methods, case definitions, follow-up, and reporting practices.

The findings in this report are subject to at least four limitations. First, the prevalence rates calculated for the newest disorders that have been added to the RUSP are based on data from only a few states across a short period, making these rates less robust than the rates for the other disorders. However, changes in estimated prevalences for these rare disorders are not likely to have a large impact on the overall rate of infants identified with an NBS disorder. Second, the study did not include two disorders on the RUSP (spinal muscular atrophy and critical congenital heart defects) because of lack of data; future studies could incorporate these disorders once reliable national data are available. Third, differences among the reporting practices of both NBS and EHDI programs potentially limit the interpretation of these data. Although NewSTEPs recommends uniform case definitions (*5*), not all NBS programs applied these definitions to the cases submitted. Finally, state prevalence estimates for individual disorders might be affected by 1) newborns born in one state and screened in another or 2) newborns from surrounding states born and screened in a state other than their resident state; however, national-level estimates would not be affected. A strength of the study is that the prior prevalence estimates of NBS disorders are modeled estimates based on four states, whereas the current study relies on reported numbers of disorders from 50 states.

The number of infants identified by NBS at a national level highlights the scope of the effect that NBS programs are having as they identify infants at risk for significant morbidity and mortality and refer them for recommended intervention. NBS continues to be a major public health achievement, offering population-based early detection, intervention, and potential improved outcomes to all infants, regardless of geographic, racial/ethnic, or socioeconomic differences (*10*).

**Summary**

**What is already known about this topic?**

Previous modeled estimates of the number of infants identified by newborn screening (NBS), in conjunction with CDC’s Hearing Screening and Follow-up Survey data, predicted approximately 10,500 cases of NBS disorders in the United States in 2006 (25.5 per 10,000 births).

**What is added by this report?**

This first national report based on reported cases from all 50 states estimates that approximately 12,900 births might be identified each year with an NBS disorder included in the study (34.0 per 10,000 births).

**What are the implications for public health practice?**

NBS continues to be one of the most successful public health interventions, offering early detection and intervention to all infants, regardless of geographic, ethnic, or socioeconomic differences.

## Footnotes

* https://www.hrsa.gov/advisory-committees/heritable-disorders/rusp/index.html.

† https://www.cdc.gov/nchs/nvss/births.htm.

§ https://www.cdc.gov/ncbddd/hearingloss/ehdi-data.html.

¶ https://www.cdc.gov/ncbddd/sicklecell/facts.html.

** https://www.hrsa.gov/sites/default/files/hrsa/advisory-committees/heritable-disorders/rusp/previous-nominations/sma-consumer-summary.pdf.

†† https://wonder.cdc.gov/.

§§ https://www.pewsocialtrends.org/2015/04/09/a-rising-share-of-the-u-s-black-population-is-foreign-born/.
